# Between-Hospital Variation in Failure to Rescue After Major Surgery

**DOI:** 10.1001/jamanetworkopen.2025.55855

**Published:** 2026-02-04

**Authors:** David Schwappach, Marcel Zwahlen, Michael M. Havranek

**Affiliations:** 1 Institute of Social and Preventive Medicine, University of Bern, Bern, Switzerland; 2 Institute of Social and Preventive Medicine, University of Bern, Bern, Switzerland; 3 Competence Center for Health Data Science, Faculty of Health Sciences and Medicine, University of Lucerne, Lucerne, Switzerland

## Abstract

**Importance:**

Failure to rescue (FTR), defined as postoperative mortality among patients with treatable complications, is a recognized patient safety concern. FTR reflects institutional capacity for timely management of deterioration and has been proposed as a quality indicator less dependent on baseline complication risk. Evidence on systematic hospital-level variation outside the US remains limited.

**Objective:**

To estimate national postoperative FTR rates, quantify between-hospital variation, and identify hospitals with better- or worse-than-expected performance using risk-standardized mortality ratios (RSMRs).

**Design, Setting, and Participants:**

This retrospective cohort study conducted in Switzerland applied the Agency for Healthcare Research and Quality (AHRQ) patient safety indicator 04 (PSI04) definition to administrative hospital data to all acute-care hospitals in Switzerland from January 2019 to December 2023. Participants included surgical inpatients with at least 1 PSI04-defined complication (ie, deep vein thrombosis and/or pulmonary embolism, pneumonia, sepsis, shock and/or cardiac arrest, and gastrointestinal hemorrhage and/or ulcer). Hospital-level variation was assessed using multilevel logistic regression with hospital random intercepts and summarized with RSMRs. Alternative models were estimated to explore the stability of results.

**Exposure:**

Acute care hospitalization.

**Main Outcomes and Measures:**

In-hospital mortality following eligible complications, expressed as crude FTR rates and RSMRs. The intraclass correlation coefficient quantified systematic performance variation.

**Results:**

Among 41 506 inpatients undergoing surgery with PSI04-defined complications (mean [SD] age, 67.6 [14.8] years; 24 692 [59.5%] men), 7310 in-hospital deaths occurred. The crude national FTR rate was 18.07 (95% CI, 17.66-18.50) of 100 admissions. In 61 hospitals with at least 100 cases, adjusted odds ratio for death varied between the lowest- and highest-performing hospitals from 0.56 (95% CI, 0.38-0.80) to 1.75 (95% CI, 1.59-1.92). Hospital-level variance was 0.114 (intraclass correlation coefficient, 0.034; 95% CI, 0.020-0.055). An estimated 1045 of 7114 observed FTR deaths (14.7%) within the hospital sample were attributable to below-average hospital performance. Five hospitals (8.2%) performed significantly better than expected, 42 (68.9%) as expected, and 14 (23.0%) substantially worse than expected based on RSMR 95% CIs. Poorer performance clustered in medium- and high-volume hospitals. Alternative regression models confirmed stability of results.

**Conclusions and Relevance:**

In this cross-sectional study of FTR, nearly 1 in 5 patients undergoing surgery who experienced serious complications died, with substantial between-hospital variation. Multilevel modeling indicated that institutional performance accounted for 1045 potentially avoidable deaths. These findings support FTR as an international patient safety indicator and highlight the need to investigate organizational determinants of variation to inform system-level improvement strategies.

## Key Points

**Question:**

What is the between-hospital variation in risk-adjusted postoperative failure-to-rescue rates in Switzerland?

**Findings:**

In this cross-sectional study of 41 506 patients including 5 years of national hospital routine data, the odds of death differed by a factor of 3.1 between the lowest- and highest-performing hospitals after accounting for measured patient-level risk factors.

**Meaning:**

These findings support positioning failure to rescue as a patient safety priority and highlight the need for deeper investigation into the practices of both underperforming and overperforming hospitals.

## Teaser

This cross-sectional study examines mortality from treatable conditions following surgery among patients in Switzerland.

## Introduction

Death from serious complications after major surgery, known as failure to rescue (FTR), remains a major patient safety concern. The term refers to the death of a patient who experiences 1 or more complications after surgery when the care team fails to respond effectively to clinical deterioration. Typically, FTR is defined by the rate of death among patients with specific postoperative complications, although definitions vary.^1^ Unlike the risk of developing complications, the risk of dying from them is thought to be less influenced by patient case mix and more reflective of institutional competencies in recognizing and managing critically illness.^2,3^ FTR is considered modifiable through improvements in postoperative care processes.^4^ In 1 US study, hospitals’ self-reported compliance with safety practices was linked to higher detection of complications but lower FTR rates.^5^ This finding suggests that complication rates may be biased by surveillance intensity, whereas FTR may better reflect performance.^6^ Recent findings indicate that in elderly adults, high postsurgical mortality is mainly driven by high FTR rates.^7^ In the United Kingdom, FTR rates have been associated with quality markers such as clinically qualified staffing per bed.^8^ US studies have linked hospital characteristics, including size and intensive care unit capacity, to better survival among patients with complications.^9^ FTR has been established as a patient safety indicator by the Agency for Healthcare Research and Quality (AHRQ), based on hospital routine data. Administrative data appeal to policymakers because they are low cost, nationally available, and generated under similar conditions across health care institutions, but they may lack clinical detail and can be affected by coding differences.^10^

For use as a cross-institutional or national safety indicator, the metric must show substantial nonrandom variation—indicating systematic performance differences—while ensuring appropriate case mix adjustment. In a systematic review, van der Linde et al^11^ found generally low hospital-level variation, lowest for clinical outcomes such as mortality and higher for process indicators. FTR rates may differ for 2 reasons: first, the impact of hospital quality on mortality may be higher than for general surgical mortality; second, FTR rates are relatively high, so even small care variations can translate to substantial effects.

In many European countries, national investigations into hospital-level FTR rates or large-scale improvement initiatives are lacking. Switzerland’s highly federalized health system delegates hospital planning and quality oversight to cantonal authorities, resulting in variation in hospital capacity and surgical case volumes. This decentralized structure may contribute to differences in hospital performance, making it important to examine variation in postoperative outcomes such as FTR. In 2019, mortality from serious postoperative complications in Switzerland was linked to an estimated excess health care cost of approximately $57 million in 2019 US dollars.^12^ Preventing deaths from treatable complications is therefore an important target from both patient safety and economic perspectives. The goal of this study was to assess the utility of FTR rates as an indicator of hospital patient safety performance in Switzerland. Specifically, we sought to (1) estimate the national postoperative FTR rate during the 5-year study period, (2) quantify between-hospital variation, and (3) identify hospitals with better- or worse-than-expected performance using risk-standardized mortality ratios (RSMRs).

## Methods

This cross-sectional study used fully anonymized national hospital data and, in accordance with the Swiss Human Research Act (Section 2, Paragraph 2c), did not require ethics committee approval or informed consent.^13^ Confidentiality and data protection agreements were signed with the Federal Statistical Office. We followed the Reporting of Studies Conducted Using Observational Routinely Collected Data (RECORD) reporting guideline, an extension of the Strengthening the Reporting of Observational Studies in Epidemiology (STROBE) guidelines.

### Data

We applied the AHRQ patient safety indicator 04 (PSI04), “death rate among surgical inpatients with serious treatable complications,” to Swiss hospitalizations from January 2019 to December 2023.^14^ The denominator includes surgical discharges of patients with eligible procedures who developed at least 1 specified complication (ie, deep vein thrombosis and/or pulmonary embolism, pneumonia, sepsis, shock and/or cardiac arrest, gastrointestinal hemorrhage and/or acute ulcer). Patients qualified if admitted electively or if the first operating room procedure code appeared within 2 days of admission. The numerator consisted of deaths among patients undergoing surgery with 1 or more of these complications. Complication-specific death rates were combined into an overall rate. PSI04 specifications were previously adapted to the Swiss medical coding system, based on AHRQ definitions, and validated in a recent record review study.^15^ The positive predictive value for the overall rate was 82% (95% CI, 77%-88%); of the 100 cases flagged in routine data, 82 were confirmed in medical records.^15^ The Swiss administrative dataset used to identify PSI04 cases^16^ contains all inpatient stays with as many as 50 diagnosis codes (*International Statistical Classification of Diseases, Tenth Revision, German Modification*),^17^ as many as 100 procedure codes (Swiss classification of surgical interventions [CHOP]),^17^ and the diagnosis-related group (SwissDRG).^18^

### Statistical Analysis

Strata-specific and overall crude yearly mortality rates were calculated to assess trends. To examine systematic differences in FTR across hospitals and over time, we estimated a multilevel logistic regression restricting the sample to hospitals with 100 cases or more during the 5-year study period (model M2). Covariates were selected a priori based on clinical plausibility, prior studies, and data availability, including potential confounders in hospital comparisons. Covariates included age, sex, insurance, Elixhauser comorbidity score, PSI04 stratum, emergency admission, and interhospital transfer. Medical disease categories (MDC) and SwissDRG were included as dummy variables. MDCs and SwissDRG variables were included if they had at least 50 PSI04 deaths, at least 15 hospitals with exposed patients, and at least 15 hospitals with both exposed and unexposed patients. In line with AHRQ, pre-MDC and infectious diseases codes were not included. Calendar year was included as a fixed effect. Hospital was modeled as a random intercept. To estimate the variation explained by differences in hospital performance, we computed the intraclass correlation coefficient (ICC).

Model performance was assessed using Brier scores.^19^ Calibration was evaluated via logistic recalibration, regressing observed outcomes on the logit-transformed estimated probabilities from the multilevel model.^20^ Hospital-level residuals were examined and correlated with case mix characteristics to assess whether unexplained variation reflected unmeasured patient factors rather than true performance differences. To estimate deaths attributable to hospital variation, we compared estimated probabilities from the full model (including hospital effects) with estimations from the same model with hospital effects set to the mean. The probability differences represented excess mortality due to hospital performance; summing these across patients yielded the total potentially attributable deaths. RSMRs for each hospital were calculated as the ratio of estimated to expected deaths, multiplied by the cohort’s national mean. We estimated 95% CIs for hospital-specific RSMRs using hierarchical bootstrap resampling with 1000 iterations. Hospitals were classified as performing better, worse, or as expected compared with the national benchmark when the 95% CI of their RSMR excluded the national rate. A funnel plot of hospitals’ RSMRs based on the observed to expected death ratio, multiplied with the national mean, was constructed with 95% and 99.8% control limits, calculated using a normal approximation to the binomial distribution. RSMRs outside this funnel were identified as hospitals with significantly divergent performance.^21,22^

To investigate the stability of results, 2 alternative models were estimated: a simple logistic model (M1), not accounting for hospital random effects, and a multilevel logistic regression with hospital random effects using the same patient-level covariates as M2, plus the hospital-level covariates log-transformed and standardized hospital surgical volume and type of hospital.^23^ Hospital performance was classified by whether the 95% CI of the RSMR covered the national crude mortality rate. Classification differences across models M1 to M3 (hospital-level covariates) were examined. Because transferred patients may present with advanced disease and bias performance comparisons, we conducted a sensitivity analysis excluding all interhospital transfers (patients transferred in before surgery).^24^ Patients transferred after surgery or transferred out were excluded per indicator definition. Using the full-sample risk model, expected mortality was estimated for nontransferred patients and RSMRs were re-estimated.

Two-sided *P* < .05 indicated statistical significance. Analyses were performed using Stata/BE, version 18.0 (StataCorp LLC).

## Results

**Figure 1.**
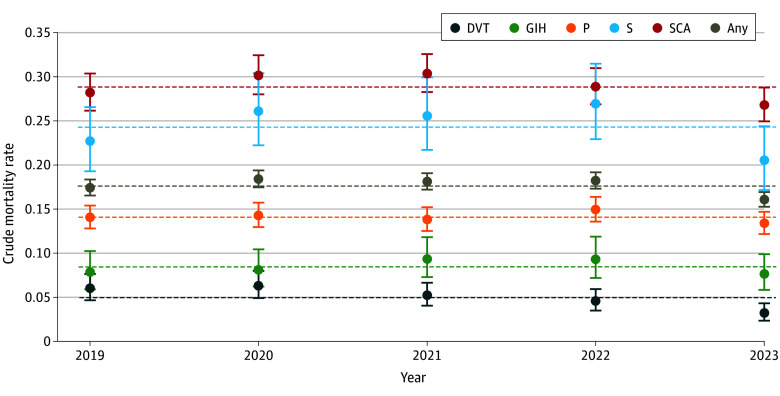
Crude Mortality Rates by Type of Complication and Year Mortality rates per 100 admissions are shown for deep vein thrombosis and/or pulmonary embolism (DVT), gastrointestinal hemorrhage and/or acute ulcer (GIH), pneumonia (P), sepsis (S), shock and/or cardiac arrest (SCA), and the overall rate (Any). Horizontal dashed lines indicate mean rates across years within each complication type.

Among 2 432 050 patients undergoing eligible procedures between 2019 and 2023, 41 506 developed any of the 5 PSI04 complications, of whom 7310 died in the hospital. Table 1 details characteristics of the PSI04 cohort. The mean (SD) age was 67.6 (14.8) years; 16 814 patients (40.5%) were female and 24 692 (59.5%) were male. Raw yearly mortality rates (Figure 1) show that deaths overall and within all strata except deep vein thrombosis rose slightly during 2020 to 2022 (ie, during the COVID-19 pandemic) before falling in 2023 to 2019 levels or lower. Of 143 hospitals treating patients in the PSI04 cohort, 61 had at least 100 cases during the 5-year study period (the hospital sample), comprising 39 360 patients and 7114 deaths (97.3% of all PSI04 deaths). The median cohort size per hospital was 278 (range, 100-3080), with a median of 48 (range, 0-806) deaths. Across the 61 hospitals, 39 360 of 1 860 623 eligible patients undergoing surgery (2.1%) experienced a PSI04-specific complication. PSI04-associated deaths accounted for 55.1% of all surgical deaths (range, 31.1%-88.9%) (eTable 1 in Supplement 1). The crude national PSI04 mortality rate among the 61 included hospitals was 18.07 (95% CI, 17.66-18.50) of 100 admissions.

**Table 1.** Death After Serious Treatable Complications (Failure to Rescue) in the Full PSI04 Cohort

Characteristic	PSI04 cohort, No. (%)^a^			
	Survived	Died	Total	*P* value^b^
All patients	34 196 (82.4)	7310 (17.6)	41 506 (100)	NA
Age, mean (SD), y	66.8 (15.1)	71.2 (12.8)	67.6 (14.8)	<.001
Sex				
Female	14 210 (41.6)	2604 (35.6)	16 814 (40.5)	<.001
Male	19 986 (58.4)	4706 (64.4)	24 692 (59.5)	
Stratum				
Deep vein thrombosis	5861 (17.1)	307 (4.2)	6168 (14.9)	<.001
Gastrointestinal hemorrhage and/or acute ulcer	3347 (9.8)	309 (4.2)	3656 (8.8)	
Pneumonia	13 377 (39.1)	2192 (30.0)	15 569 (37.5)	
Sepsis	2378 (7.0)	763 (10.4)	3141 (7.6)	
Shock and/or cardiac arrest	9233 (27.0)	3739 (51.1)	12 972 (31.3)	
Admitted by hospital transfer				
No	30 820 (90.1)	5965 (81.6)	36 785 (88.6)	<.001
Yes	3376 (9.9)	1345 (18.4)	4721 (11.4)	
Emergency admission				
No	18 090 (52.9)	2429 (33.2)	20 519 (49.4)	<.001
Yes	16 106 (47.1)	4881 (66.8)	20 987 (50.6)	
Private insurance				
No	27 026 (79.0)	5942 (81.3)	32 968 (79.4)	<.001
Yes	7170 (21.0)	1368 (18.7)	8538 (20.6)	
Major diagnostic category				
Nervous system	1870 (5.5)	766 (10.5)	2636 (6.4)	<.001
Respiratory system	474 (1.4)	126 (1.7)	600 (1.4)	
Circulatory system	5527 (16.2)	1027 (14.0)	6554 (15.8)	
Digestive system	5113 (15.0)	740 (10.1)	5853 (14.1)	
Hepatobiliary system and pancreas	1112 (3.3)	134 (1.8)	1246 (3.0)	
Musculoskeletal system and connective tissue	5913 (17.3)	500 (6.8)	6413 (15.5)	
Kidney and urinary tract	1261 (3.7)	106 (1.5)	1367 (3.3)	
Other infectious and parasitic diseases	2005 (5.9)	767 (10.5)	2772 (6.7)	
Major trauma	437 (1.3)	67 (0.9)	504 (1.2)	
Other	4015 (11.7)	270 (3.7)	4285 (10.3)	
Pre-MDC (transplants, intensive care, etc)	6469 (18.9)	2807 (38.4)	9276 (22.3)	
SwissDRG				
Complex craniotomy or spinal surgery	441 (1.3)	270 (3.7)	711 (1.7)	<.001
Complex procedures on nervous system	445 (1.3)	296 (4.0)	741 (1.8)	
Complex procedures on respiratory system	158 (0.5)	87 (1.2)	245 (0.6)	
PTCA	938 (2.7)	96 (1.3)	1034 (2.5)	
Complex procedures on cardiovascular system	1202 (3.5)	554 (7.6)	1756 (4.2)	
Procedures on cardiovascular system	463 (1.4)	88 (1.2)	551 (1.3)	
Complex vascular procedures	518 (1.5)	56 (0.8)	574 (1.4)	
Other complex procedures on digestive organs	278 (0.8)	65 (0.9)	343 (0.8)	
Other complex procedures on intestine or enterostomy	448 (1.3)	59 (0.8)	507 (1.2)	
Procedures on small or large intestine, stomach, esophagus, duodenum	839 (2.5)	72 (1.0)	911 (2.2)	
Complex procedures on digestive organs	1077 (3.1)	336 (4.6)	1413 (3.4)	
Other procedures on hip joint and femur	799 (2.3)	160 (2.2)	959 (2.3)	
Implantation, replacement, or revision of hip endoprosthesis	1187 (3.5)	116 (1.6)	1303 (3.1)	
Procedures for infectious and parasitic diseases, including sepsis	1193 (3.5)	472 (6.5)	1665 (4.0)	
Complex treatment for infectious and parasitic diseases	507 (1.5)	248 (3.4)	755 (1.8)	
Other or unclassified	18 209 (53.2)	1648 (22.5)	19 857 (47.8)	
Pre-MDC (transplants, intensive care, etc)	5494 (16.1)	2687 (36.8)	8181 (19.7)	
Elixhauser comorbidity index, mean (SD)	4.0 (2.4)	5.0 (2.4)	4.1 (2.4)	<.001
Length of stay, mean (SD), d	20.8 (19.8)	16.9 (22.5)	20.2 (20.3)	<.001
Hospital type				
University	10 669 (31.2)	2757 (37.7)	13 426 (32.4)	<.001
Cantonal center	19 300 (56.4)	4085 (55.9)	23 385 (56.3)	
Regional or specialized	3950 (11.6)	424 (5.8)	4374 (10.5)	
Other	277 (0.8)	44 (0.6)	321 (0.8)	

Abbreviations: MDC, medical disease category; NA, not applicable; PTCA, percutaneous transluminal coronary angioplasty.

^a^ Percentages for the first row are row percentages; remaining are column percentages.

^b^ Calculated using 2-sample 2-sided *t* tests for continuous, 2-sided Pearson χ^2^ tests for categorical variables.

Table 2 shows multilevel logistic regression results. Calendar year was associated with mortality (Wald χ^2^_4_ = 23.27; *P* < .001). Compared with 2019, odds were slightly higher in 2021 (odds ratio [OR], 1.10; 95% CI, 1.01-1.21; *P* = .03) and slightly lower in 2023 (OR, 0.91; 95% CI, 0.83-0.99; *P* = .04). A joint Wald test of hospital-by-year interaction terms indicated no statistically significant variation in time trends across hospitals (χ^2^_224_ = 226.24; *P* = .45), supporting a model with year as a fixed effect.

**Table 2.** Failure to Rescue, Multilevel Logistic Regression Model^a^

Covariate	Odds ratio (95% CI)^b^	*P* value
Year		
2019	1 [Reference]	NA
2020	1.08 (0.99-1.18)	.09
2021	1.10 (1.01-1.21)	.03
2022	1.05 (0.96-1.15)	.27
2023	0.91 (0.83-0.99)	.04
Age group, y		
<45	1 [Reference]	NA
46-65	1.74 (1.52-1.99)	<.001
66-75	2.43 (2.12-2.78)	<.001
76-85	3.99 (3.48-4.57)	<.001
>85	6.05 (5.13-7.12)	<.001
Male sex	1.08 (1.01-1.14)	.02
Private insurance	0.84 (0.78-0.90)	<.001
Elixhauser comorbidity index, mean	1.04 (1.03-1.05)	<.001
Stratum		
Deep vein thrombosis	1 [Reference]	NA
Gastrointestinal hemorrhage and/or acute ulcer	1.49 (1.25-1.77)	<.001
Pneumonia	2.00 (1.75-2.29)	<.001
Sepsis	3.85 (3.30-4.49)	<.001
Shock and/or cardiac arrest	5.76 (5.02-6.62)	<.001
Emergency admission	1.66 (1.56-1.77)	<.001
Admitted by hospital transfer	1.56 (1.44-1.69)	<.001
Medical disease categories		
Nervous system	1.83 (1.45-2.32)	<.001
Respiratory system	2.35 (1.57-3.51)	<.001
Circulatory system	1.22 (0.98-1.53)	.08
Digestive system	0.97 (0.77-1.23)	.82
Hepatobiliary system	1.24 (0.96-1.60)	.10
Musculoskeletal system	0.89 (0.71-1.12)	.32
Kidney and urinary system	0.93 (0.71-1.21)	.58
Major trauma	2.18 (1.58-3.01)	<.001
Other	0.89 (0.72-1.11)	.30
SwissDRG		
Complex craniotomy or spinal surgery	1.04 (0.78-1.40)	.77
Complex procedures on nervous system	1.20 (0.90-1.60)	.20
Complex procedures on respiratory system	1.04 (0.63-1.69)	.89
PTCA	0.29 (0.21-0.40)	<.001
Complex procedures on cardiovascular system	1.42 (1.11-1.83)	.006
Procedures on cardiovascular system	0.64 (0.46-0.89)	.008
Complex vascular procedures	0.42 (0.29-0.61)	<.001
Other complex procedures on digestive organs	0.73 (0.50-1.07)	.11
Other complex procedures on intestine or enterostomy	0.41 (0.28-0.60)	<.001
Procedures on small or large intestine, stomach, esophagus, duodenum	0.32 (0.23-0.46)	<.001
Complex procedures on digestive organs	0.65 (0.50-0.86)	.002
Implantation, replacement, or revision of hip endoprosthesis	0.44 (0.33-0.61)	<.001
Other or unclassified	0.33 (0.27-0.39)	<.001
Between-hospital variance	0.11 (0.07-0.19)	<.001

Abbreviations: NA, not applicable; PTCA, percutaneous transluminal coronary angioplasty.

^a^ Includes 39 360 observations from 61 hospitals.

^b^ Shown for fixed effects. Random-effect variance is reported in variance units.

A likelihood ratio test comparing this model with a single-level logistic regression showed a significant improvement in fit (χ^2^_1_ = 217.87; *P* < .001), confirming that between-hospital variability was nonnegligible. The random intercept variance at the hospital level was estimated at 0.11 (95% CI, 0.07-0.19). The ICC of 0.034 (95% CI, 0.020-0.055) indicated that approximately 3.4% of the total variance in mortality was attributable to differences between hospitals, whereas 96.6% was due to patient-level differences or other unmeasured factors. The distribution of hospital-specific random intercepts suggests meaningful between-hospital variation in risk-adjusted outcome probabilities. The adjusted ORs ranged from 0.56 (95% CI, 0.38-0.80) to 1.75 (95% CI, 1.59-1.92 [ie, a factor of 3.1]).

The model demonstrated acceptable discrimination, with an area under the receiver operating characteristics curve of 0.79 (95% CI, 0.79-0.80). Calibration was further assessed using logistic recalibration, regressing observed outcomes on the logit-transformed estimated probabilities. The estimated calibration slope was 1.00 (95% CI, 0.98-1.04), indicating accurate estimations across the risk spectrum, and the intercept was 0.01 (*P* = .26), indicating good calibration in the large. The Brier score was 0.12, suggesting good overall accuracy. Spiegelhalter *z* statistic also showed no evidence of miscalibration (*z* = 1.23; *P* = .11). Mean estimated mortality (18.04 per 100 admissions) closely matched observed mortality (18.07 per 100 admissions). Hospital-level residuals were symmetrically distributed around zero with no extreme outliers; all standardized random effects fell within 2 SDs. However, residual variation confirmed that meaningful hospital-level variation remained unexplained by patient-level factors. Hospital-level regression of case mix variables (eg, the proportion of patients within each stratum) on hospital residuals revealed no associations except for hospital transfer. The proportion of patients received via hospital transfer correlated with hospital-level residuals (*r* = 0.52; *P* < .001).

**Figure 2.**
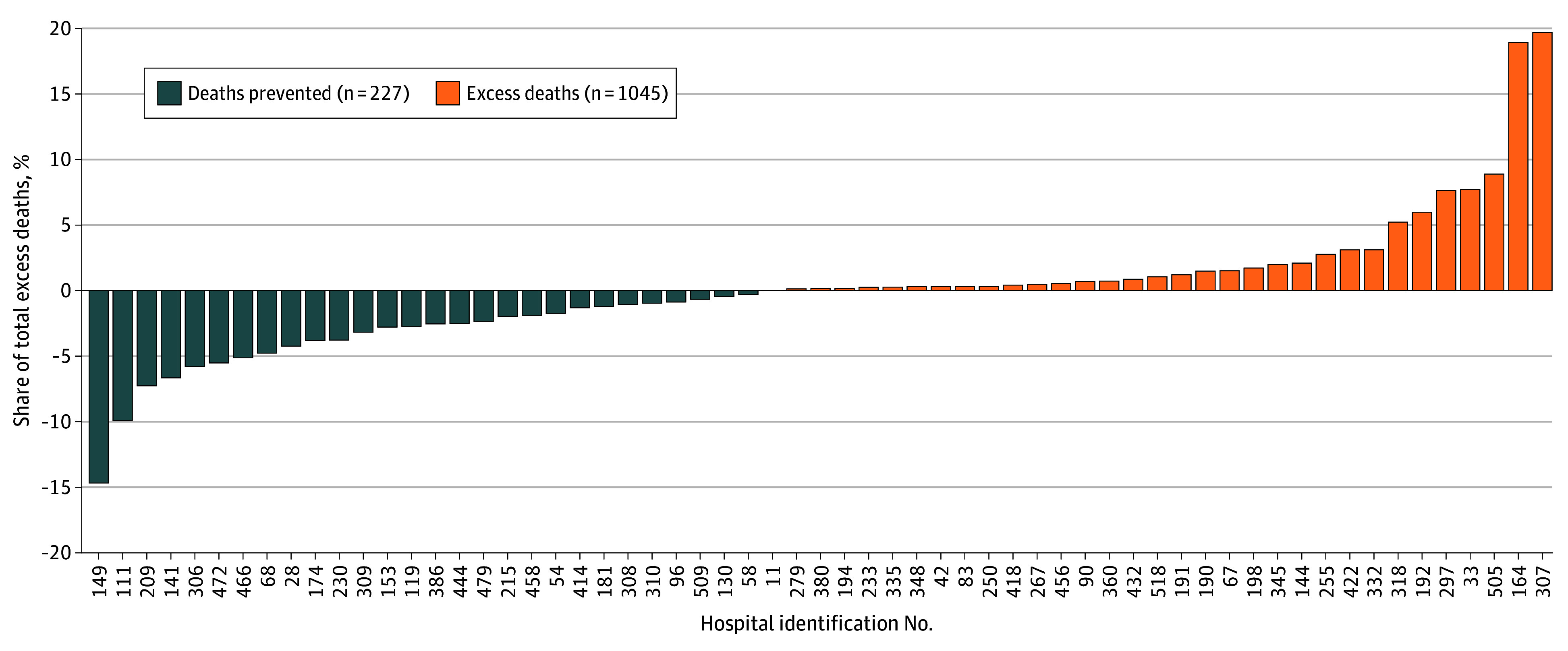
Positive and Negative Excess Deaths by Hospital Each bar represents the proportion of positive excess deaths (patients who died due to worse-than-expected performance) or negative excess deaths (patients who survived due to better-than-expected performance) for that hospital, expressed relative to the total number of positive or negative excess deaths across all hospitals.

**Figure 3.**
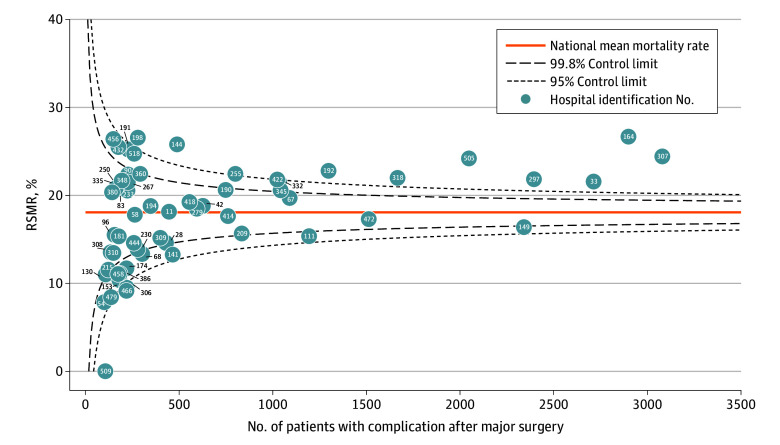
Funnel Diagram of Risk-Standardized Mortality Rates (RSMR) by Hospital Each circle represents a hospital’s RSMR, calculated as observed divided by expected mortality and multiplied by the national mean, plotted against the number of patients with complications after major surgery. Funnel lines show 95% and 99.8% control limits of the national mortality rate. Hospital 509 had no deaths and is shown for completeness only.

Five hospitals (8.2%) performed significantly better than expected, 42 (68.9%) performed as expected, and 14 (23.0%) performed substantially worse than expected based on RSMR 95% CIs (M2 estimates in eTable 2 in Supplement 1). The number of deaths potentially attributable to inferior hospital performance, defined as excess deaths compared with the national mean hospital risk, was estimated at 1045 during the 5-year period (14.7% of 7114 observed PSI04 deaths in the sample). A large fraction of the positive excess deaths concentrated in a few hospitals (Figure 2); 2 hospitals accounted for 39% of all excess deaths. Figure 3 presents a funnel plot of hospitals’ RSMRs (M2: [observed deaths/expected deaths] × national mean), plotted against case load. Of the 61 hospitals included, 30 (49.2%) had risk-standardized mortality rates as expected, 14 (23.0%) performed better than expected, and 17 (27.9%) performed worse than expected (based on their position relative to the 95% CI of the national mortality rate).

For the regression model with added hospital-level covariates (M3), only log standardized surgical volume was statistically significant (OR, 1.13; 95% CI, 1.03-1.24; *P* = .008), while hospital type for all categories was not. As expected, RSMR variability decreased substantially across models due to hierarchical shrinkage, with SDs of 4.96 in M1, 3.87 in M2, and 2.83 in M3 (eTable 2 in Supplement 1). Hospitals’ RSMRs were highly correlated among the 3 models (M1-M2: *r* = 0.95 [*P* < .001]; M2-M3: *r* = 0.81 [*P* < .001]; M1-M3: *r* = 0.79 [*P* < .001]). For 32 hospitals, the performance classification was unaffected by model choice.

Patients who transferred in (n = 4564) were younger (mean [SD] age, 65.8 [14.8] vs 67.7 [14.8] years; *P* < .001), had higher Elixhauser scores (mean [SD], 4.7 [2.4] vs 4.1 [2.4]; *P* < .001), and more frequently developed shock (1637 of 4564 [35.9%] vs 10 884 of 34 796 [31.3%]; *P* < .001) than nontransferred patients. Transfers were concentrated in higher-mortality hospitals: the 5 hospitals receiving the largest proportions (8.2%-17.7%) all had RSMRs above expected. RSMRs calculated with and without transferred patients were highly correlated (*r* = 0.99; *P* < .001), but absolute differences were substantial (mean difference, 1.90; range, 0.94-4.05).

## Discussion

In this cross-sectional study of inpatients undergoing surgery in Switzerland, nearly one-fifth of patients developing postoperative complications died. Internationally, reported FTR rates range from 1% to more than 40%, depending on definitions and populations.^25^ Using the AHRQ PSI04 metric, US hospitals had an estimated rate of 15.2 FTR deaths per 100 discharges,^26^ whereas Belgium reported 22.6 per 100.^27^ This suggests that, despite structural and organizational differences between the Swiss and US health systems, the overall capacity to prevent deaths following major postoperative complications is broadly similar. However, we observed substantial between-hospital variation: odds of death varied 3.1-fold between the lowest- and highest-performing hospitals after adjusting for patient-level risk. Although the hospital-level variance was moderate (ICC = 0.034), excess deaths attributable to performance differences represented 15% of observed deaths, underscoring that even modest variance can translate into a considerable number of potentially avoidable deaths in a large patient population with a substantial baseline risk.

The Swiss health system is highly decentralized. Each of the 26 cantons in Switzerland plans hospital capacity and regulates quality oversight with limited national coordination. National centralization policies exist for highly specialized procedures but affect only a small subset of surgical care.^28^ Cantonal capacity plans sometimes set minimum volume requirements that vary across cantons, are commonly not evidence based, and are often unmet. Quality improvement initiatives are primarily locally driven and voluntary. This structural heterogeneity may contribute to interhospital differences in resources, staffing, and escalation processes. We observed substantial FTR variation between hospitals even after accounting for patient-level risk, highlighting how decentralized governance can lead to meaningful differences in patient outcomes.

Our model demonstrated good performance at the national level; however, the concentration of higher RSMRs in high-volume hospitals was unexpected. In our model including hospital-level covariates (M3), risk of death significantly increased with overall surgical volume, thereby confirming these findings. Previous studies generally associated larger hospital size with lower FTR rates, potentially due to greater procedural experience and enhanced capability to manage clinical deterioration.^9,29,30,31^ Several factors may explain our findings. Residual confounding may persist if case complexity or proportion of high-risk interventions is incompletely captured. Diseconomies of scale could emerge in high-volume settings, whereby organizational complexity impairs communication and coordination.^32^ High-volume hospitals also had greater proportions of transferred-in patients with higher comorbidity burdens and shock, which are characteristics associated with mortality risk. However, transfer rates were moderately correlated with unexplained hospital-level variance. Excluding transferred patients substantially decreased RSMRs in hospitals with high transfer proportions but minimally affected relative RSMR ordering, suggesting poor performance is not directly explained by transferred patients. Instead, transfer rates may serve as an institutional marker for complex coordination demands or resource strain influencing outcomes across patient populations. Our results emphasize that in-depth analysis of the microsystem characteristics is required for hospitals’ performance managing complications relative to hospital volume.^9^

### Limitations

A key limitation of this analysis lies in the routine hospital data, where 3 sources of variation meld, including unadjusted differences in cases-mix, differences in coding practices, and true differences in hospital performance, and there is no reliable way to disentangle them. More granular risk-adjustment might better capture severity, including within specific complication strata. Although no evidence of bias was identified, interhospital differences in coding practices cannot be excluded. The PSI04 denominator depends on accurate recording of postoperative complications, yet coding behavior may vary systematically. University or tertiary centers may preferentially code severe, high-acuity events, while smaller or resource-limited hospitals may fail to detect or underreport complications, particularly when rapid deterioration progresses quickly to death. Such inconsistencies would produce artificial variation in FTR rates.

The AHRQ definition of FTR has been critiqued as a hospital safety measure for 2 principal reasons. First, the inclusion of bedside procedures such as bronchoscopy or angiography—often performed by nonsurgeons—raises questions about its framing as surgical quality.^33^ However, this does not preclude its utility as a broader patient safety indicator, as it may capture institutional capacities for cross-disciplinary collaboration, timely recognition of complications, and effective rescue responses. Second, concerns about accuracy stem from the inclusion of complications present on admission, which reverses the presumed causal sequence and may disproportionately affect high-acuity centers.^34^ For example, in a US trauma center review,^35^ 6 of 11 flagged cases were confirmed, all clinically meaningful, although only one revealed opportunities for improvement. In contrast, another retrospective review reported a positive predictive value of 100%.^36^ In Switzerland, validation of the indicator showed an overall positive predictive value of 82%; in two-thirds of false-positive findings, events were present on admission.^15^ Enhancing coding practices and refining indicator definitions will further improve validity.^37,38^

In-depth examination of high-performing (ie, positively deviant) hospitals could generate hypotheses about mechanisms of their superior performance,^39^ such as earlier recognition, more effective escalation, or faster intervention in patients with deteriorating conditions^40^ and identify whether performance differences are concentrated in specific interventions, wards, or patient populations, as well as the contributing organizational factors. Finally, the level of between-hospital variation in FTR rates could be examined for procedures with differing degrees of centralization and referral patterns to determine whether more deliberately centralized services are associated with lower variability.^40^

### Conclusions

In this cross-sectional study of patients in Switzerland who underwent surgery and experienced postoperative complications, we found substantial variation in FTR across hospitals. Our findings support prioritizing FTR as a national patient safety objective. Future research should evaluate why some hospitals outperform others and what factors at the hospital, ward, procedure, and patient level are associated with FTR. Our analyses provide several angles for further research and targeted improvement activities that may help to improve the safety of care of patients undergoing surgery.

## Notes

Supplement 1. **eTable 1.** Overall and PSI04-Related Surgical Deaths in the Hospital Sample (n = 61), 2019 to 2023 **eTable 2.** Risk-Standardized Mortality Rates (RSMR) by Hospital Across 3 Regression Models (61 Hospitals)

Supplement 2.
Data Sharing Statement
