# Supplementary material for: Between-Hospital Variation in Failure to Rescue After Major Surgery
Source: JAMA Netw Open. 2026 Feb 4;9(2):e2555855. doi: 10.1001/jamanetworkopen.2025.55855 (PMC12873769; doi:10.1001/jamanetworkopen.2025.55855)
Supplement: Supplement 1. — eTable 1. Overall and PSI04-Related Surgical Deaths in the Hospital Sample (n = 61), 2019 to 2023 eTable 2. Risk-Standardized Mortality Rates (RSMR) by Hospital Across 3 Regression Models (61 Hospitals) [file jamanetwopen-e2555855-s001.pdf]

Supplementary Online Content

Schwappach D, Zwahlen M, Havranek MM. Between-hospital variation in failure to rescue after major surgery. *JAMA Netw Open.* 2026;9(2):e2555855.
doi:10.1001/jamanetworkopen.2025.55855

eTable 1. Overall and PSI04-Related Surgical Deaths in the Hospital Sample (n = 61), 2019 to 2023

eTable 2. Risk-Standardized Mortality Rates (RSMR) by Hospital Across 3 Regression Models (61 Hospitals)

This supplementary material has been provided by the authors to give readers additional information about their work.

© 2026 Schwappach D et al. *JAMA Network Open*.

eTable 1. Overall and PSI04-Related Surgical Deaths in the Hospital Sample (n = 61), 2019 to 2023

Hospital ID	N surgical patients	N surgical deaths	N PSI04 patients	N PSI04 deaths	Surgical Mortality rate, %	Surgical Mortality rate lower CI	Surgical Mortality rate upper CI	PSI04 Mortality rate, %	PSI04 Mortality rate lower CI	PSI04 Mortality rate upper CI	Share PSI04 deaths / all deaths, %
11	17540	137	446	70	0.78	0.66	0.92	15.70	12.24	19.83	51.09
28	18276	77	429	48	0.42	0.33	0.53	11.19	8.25	14.83	62.34
33	54990	869	2713	510	1.58	1.48	1.69	18.80	17.20	20.50	58.69
42	33916	205	626	98	0.60	0.52	0.69	15.65	12.71	19.08	47.80
54	4816	11	100	6	0.23	0.11	0.41	6.00	2.20	13.06	54.55
58	15783	63	263	46	0.40	0.31	0.51	17.49	12.81	23.33	73.02
67	48759	367	1090	205	0.75	0.68	0.83	18.81	16.32	21.57	55.86
68	22273	65	302	38	0.29	0.23	0.37	12.58	8.90	17.27	58.46
83	11371	80	188	36	0.70	0.56	0.88	19.15	13.41	26.51	45.00
90	15304	67	228	49	0.44	0.34	0.56	21.49	15.90	28.41	73.13
96	13887	29	155	18	0.21	0.14	0.30	11.61	6.88	18.35	62.07
111	47344	287	1193	136	0.61	0.54	0.68	11.40	9.56	13.48	47.39
119	8100	18	132	16	0.22	0.13	0.35	12.12	6.93	19.68	88.89
130	24810	6	107	4	0.02	0.01	0.05	3.74	1.02	9.57	66.67
141	19184	75	465	49	0.39	0.31	0.49	10.54	7.80	13.93	65.33
144	57845	163	489	86	0.28	0.24	0.33	17.59	14.07	21.72	52.76
149	61405	569	2340	333	0.93	0.85	1.01	14.23	12.74	15.84	58.52
153	13364	21	181	13	0.16	0.10	0.24	7.18	3.82	12.28	61.90
164	85852	1311	2899	629	1.53	1.45	1.61	21.70	20.03	23.46	47.98
174	18535	34	219	22	0.18	0.13	0.26	10.05	6.30	15.21	64.71
181	14254	38	178	22	0.27	0.19	0.37	12.36	7.75	18.71	57.89
190	33943	248	748	140	0.73	0.64	0.83	18.72	15.74	22.09	56.45
191	14579	77	228	58	0.53	0.42	0.66	25.44	19.32	32.89	75.32
192	47403	641	1297	317	1.35	1.25	1.46	24.44	21.82	27.29	49.45

© 2026 Schwappach D et al. *JAMA Network Open*.

194	19201	82	348	59	0.43	0.34	0.53	16.95	12.91	21.87	71.95
198	33894	158	280	70	0.47	0.40	0.54	25.00	19.49	31.59	44.30
209	22658	221	834	116	0.98	0.85	1.11	13.91	11.49	16.68	52.49
215	7166	27	122	13	0.38	0.25	0.55	10.66	5.67	18.22	48.15
230	16187	52	278	36	0.32	0.24	0.42	12.95	9.07	17.93	69.23
233	25226	70	224	32	0.28	0.22	0.35	14.29	9.77	20.17	45.71
250	15755	59	176	33	0.37	0.29	0.48	18.75	12.91	26.33	55.93
255	62542	345	800	162	0.55	0.49	0.61	20.25	17.25	23.62	46.96
267	16033	57	225	43	0.36	0.27	0.46	19.11	13.83	25.74	75.44
279	82405	161	594	84	0.20	0.17	0.23	14.14	11.28	17.51	52.17
297	66729	847	2394	479	1.27	1.19	1.36	20.01	18.26	21.88	56.55
306	10594	33	221	20	0.31	0.21	0.44	9.05	5.53	13.98	60.61
307	78071	1283	3080	806	1.64	1.55	1.74	26.17	24.39	28.04	62.82
308	14186	29	134	12	0.20	0.14	0.29	8.96	4.63	15.64	41.38
309	27995	97	400	45	0.35	0.28	0.42	11.25	8.21	15.05	46.39
310	24890	22	149	11	0.09	0.06	0.13	7.38	3.69	13.21	50.00
318	77523	672	1666	320	0.87	0.80	0.93	19.21	17.16	21.43	47.62
332	41235	316	1032	217	0.77	0.68	0.86	21.03	18.32	24.02	68.67
335	8577	58	162	32	0.68	0.51	0.87	19.75	13.51	27.89	55.17
345	52492	357	1041	185	0.68	0.61	0.75	17.77	15.30	20.52	51.82
348	15469	52	193	29	0.34	0.25	0.44	15.03	10.06	21.58	55.77
360	40588	115	292	51	0.28	0.23	0.34	17.47	13.00	22.96	44.35
380	13655	43	141	24	0.31	0.23	0.42	17.02	10.91	25.33	55.81
386	22059	45	183	14	0.20	0.15	0.27	7.65	4.18	12.84	31.11
414	33642	205	760	122	0.61	0.53	0.70	16.05	13.33	19.17	59.51
418	19925	122	555	86	0.61	0.51	0.73	15.50	12.39	19.14	70.49
422	31040	361	1024	204	1.16	1.05	1.29	19.92	17.28	22.85	56.51

© 2026 Schwappach D et al. *JAMA Network Open*.

432	20408	84	176	43	0.41	0.33	0.51	24.43	17.68	32.91	51.19
444	15419	53	258	31	0.34	0.26	0.45	12.02	8.16	17.06	58.49
456	21178	42	149	28	0.20	0.14	0.27	18.79	12.49	27.16	66.67
458	17887	24	174	11	0.13	0.09	0.20	6.32	3.16	11.31	45.83
466	17030	25	219	17	0.15	0.10	0.22	7.76	4.52	12.43	68.00
472	56620	517	1512	288	0.91	0.84	1.00	19.05	16.91	21.38	55.71
479	6408	17	136	8	0.27	0.15	0.42	5.88	2.54	11.59	47.06
505	68015	726	2047	381	1.07	0.99	1.15	18.61	16.79	20.58	52.48
509	36295	0	106	0	0.00	0.00	0.01	0.00	0.00	3.48	.
518	18093	78	259	53	0.43	0.34	0.54	20.46	15.33	26.77	67.95

PSI04 includes deep vein thrombosis/pulmonary embolism, gastrointestinal hemorrhage/acute ulcer, pneumonia, sepsis, shock/cardiac arrest
Mortality rates are unadjusted, crude rates.
“lower CI” and “upper CI” stand for the lower and upper end of the 95% confidence interval
Hospital 509 reports no surgical deaths.

© 2026 Schwappach D et al. *JAMA Network Open*.

eTable 2. Risk-Standardized Mortality Rates (RSMR) by Hospital Across 3 Regression Models (61 Hospitals)

Hospital ID	M1 RSMR	M1 CI Lower	M1 CI Upper	M2 RSMR	M2 CI Lower	M2 CI Upper	M3 RSMR	M3 CI Lower	M3 CI Upper	M1 Class	M2 Class	M3 Class
11	16.17	12.97	19.43	18.08	15.30	21.07	18.40	15.77	20.74	2	2	2
28	13.08	9.79	16.53	15.15	13.07	18.92	15.65	13.97	19.01	1	2	2
33	18.85	17.50	20.24	21.48	19.86	23.02	18.20	16.95	19.55	2	3	2
42	17.10	14.33	20.21	18.69	16.04	21.50	17.51	15.40	19.98	2	2	2
54	7.26	2.26	13.76	12.90	12.93	19.23	16.18	14.80	21.40	1	2	2
58	16.34	12.11	20.30	17.81	15.02	21.19	18.30	15.67	20.85	2	2	2
67	17.37	15.50	19.33	19.59	17.54	21.39	17.45	15.91	19.10	2	2	2
68	12.22	8.84	15.81	14.28	12.70	18.22	14.65	13.78	18.36	1	2	2
83	19.12	13.98	24.57	19.95	16.01	23.24	23.59	17.10	24.27	2	2	2
90	20.39	15.95	25.01	21.34	17.04	24.34	21.49	17.03	23.14	2	2	2
96	14.28	8.53	20.97	16.37	13.65	20.89	17.22	14.93	20.37	2	2	2
111	13.89	11.89	15.90	15.53	13.69	17.78	14.01	12.92	16.31	1	1	1
119	10.42	6.06	15.58	13.69	12.84	18.81	16.91	15.00	20.81	1	2	2
130	9.68	2.18	20.73	15.34	14.18	21.72	16.95	15.14	21.86	2	2	2
141	12.03	9.09	15.08	13.99	12.32	17.78	14.51	13.28	18.08	1	1	2
144	22.93	19.00	26.99	24.64	19.67	27.16	21.04	17.45	22.93	3	3	2
149	14.60	13.20	16.02	16.43	15.11	18.09	13.82	12.98	15.50	1	2	1
153	9.25	4.69	13.88	13.03	12.11	18.90	16.04	14.36	21.09	1	2	2
164	22.59	21.21	24.07	26.45	24.62	27.72	21.00	19.59	22.03	3	3	3
174	10.65	6.54	14.68	13.47	12.19	18.04	14.35	13.69	18.53	1	1	2
181	14.05	9.44	19.15	16.17	13.89	20.53	18.01	15.55	20.95	2	2	2
190	17.97	15.50	20.29	20.36	17.60	22.70	19.00	16.66	21.01	2	2	2
191	23.17	18.62	27.91	23.57	18.50	25.75	23.54	18.02	24.34	3	3	2
192	19.91	17.94	21.79	22.57	20.49	23.96	20.16	18.36	21.30	2	3	3
194	16.97	13.36	20.33	18.61	15.65	21.42	18.66	16.00	20.97	2	2	2

© 2026 Schwappach D et al. *JAMA Network Open*.

198	23.09	19.20	27.24	24.89	19.69	27.09	22.76	18.25	23.77	3	3	3
209	14.07	11.95	16.34	15.85	13.98	18.49	15.78	14.17	18.25	1	2	2
215	10.49	5.73	16.05	14.11	13.15	19.52	17.38	15.02	21.45	1	2	2
230	12.73	9.09	16.54	14.76	12.92	18.57	15.55	14.16	19.00	1	2	2
233	18.73	13.23	24.12	19.76	15.67	22.98	19.07	15.96	21.20	2	2	2
250	19.10	13.96	25.11	20.17	15.98	23.54	20.27	16.26	22.40	2	2	2
255	19.67	17.21	22.20	22.07	19.25	24.12	18.92	16.86	20.69	2	3	2
267	19.62	14.99	24.36	20.59	16.47	23.61	20.69	16.57	22.44	2	2	2
279	16.75	13.65	19.61	18.38	15.56	21.36	15.56	13.99	18.45	2	2	2
297	19.00	17.65	20.49	21.71	20.09	23.07	17.94	16.72	19.16	2	3	2
306	8.72	5.51	12.40	11.84	11.43	16.86	14.96	14.18	19.66	1	1	2
307	20.95	19.82	22.05	24.31	22.88	25.46	19.82	18.71	20.75	3	3	3
308	12.24	6.05	19.00	15.38	13.48	20.87	17.91	15.14	22.16	2	2	2
309	13.89	10.45	17.38	15.65	13.46	19.25	15.41	14.04	18.76	1	2	2
310	12.05	6.22	18.52	15.40	13.70	21.01	17.26	14.88	21.58	2	2	2
318	19.39	17.64	21.20	21.82	19.74	23.41	18.13	16.59	19.55	2	3	2
332	19.53	17.46	21.61	21.30	18.86	23.28	19.32	17.26	21.05	2	3	2
335	18.86	13.73	24.49	19.90	15.84	23.16	21.10	16.19	22.65	2	2	2
345	18.32	16.04	20.58	20.39	18.01	22.52	17.89	16.15	19.76	2	2	2
348	19.87	13.55	25.89	20.45	15.91	23.70	23.14	16.97	24.10	2	2	2
360	20.23	15.59	25.45	21.44	17.17	24.29	19.51	16.44	21.69	2	2	2
380	18.37	12.29	25.07	19.45	15.35	22.74	21.98	16.25	23.27	2	2	2
386	10.09	5.64	15.18	13.52	12.55	19.17	15.81	14.40	20.64	1	2	2
414	15.85	13.43	18.28	17.64	15.49	20.18	16.61	14.93	18.93	2	2	2
418	17.45	14.29	20.80	19.05	16.30	21.81	19.02	16.45	21.16	2	2	2
422	19.33	17.36	21.63	21.54	19.00	23.52	20.25	17.91	21.92	2	3	2
432	22.53	16.77	28.28	23.40	17.76	25.88	25.56	17.95	25.50	2	2	2

© 2026 Schwappach D et al. *JAMA Network Open*.

444	13.14	9.17	17.37	15.38	13.29	19.67	16.21	14.48	19.90	1	2	2
456	24.17	16.24	31.44	23.36	16.84	25.73	22.16	16.59	22.84	2	2	2
458	9.39	4.49	14.64	13.77	12.69	19.80	16.32	14.60	21.15	1	2	2
466	8.38	5.21	11.92	11.81	11.47	17.14	13.06	13.54	17.86	1	1	1
472	15.56	13.96	17.12	17.32	15.84	18.98	15.22	14.24	16.80	1	2	1
479	7.34	2.82	12.64	12.47	12.30	19.12	16.03	14.52	21.35	1	2	2
505	20.80	19.00	22.52	23.97	21.79	25.63	20.12	18.43	21.48	3	3	3
509	0.00	0.00	0.00	12.54	13.78	20.82	14.60	14.66	21.73	1	2	2
518	22.56	17.44	27.88	23.21	18.16	25.86	22.77	17.55	23.95	2	3	2

RSMR = Risk-Standardized Mortality Rate (per 100 patients). CI lower / upper refer to bootstrapped 95% confidence intervals.
Model 1 (M1): Simple logistic regression. RSMR= Observed/Expected deaths * national crude mortality rate.
Model 2 (M2): Hierarchical model with patient-level risk adjustment only (see table 2). RSMR= Predicted/Expected deaths * national crude mortality rate.
Model 3 (M3): Hierarchical model with patient-level risk adjustment plus log-transformed and standardized hospital surgical volume and type of hospital (university / large cantonal / regional & specialized clinics). RSMR= Predicted/Expected deaths * national crude mortality rate.
For M1, we implemented case-resampling bootstrap, resampling patients with replacement within each hospital to preserve the clustering structure.
For M2 and M3, we used hierachical parametric bootstrap procedures.
Classification based on whether hospital 95% CI covers national crude mortality rate (18.07), with 1=Better 2=Expected 3=Worse.
“lower CI” and “upper CI” stand for the lower and upper end of the 95% confidence interval
Hospital 509 reports no surgical deaths

© 2026 Schwappach D et al. *JAMA Network Open*.
