# Supplementary material for: Between-Hospital Variation in Failure to Rescue After Major Surgery
Source: JAMA Netw Open. 2026 Feb 4;9(2):e2555855. doi: 10.1001/jamanetworkopen.2025.55855 (PMC12873769; doi:10.1001/jamanetworkopen.2025.55855)
Supplement: Supplement 2. — Data Sharing Statement [file jamanetwopen-e2555855-s002.pdf]

Data Sharing Statement

Schwappach. Between-Hospital Variation in Failure to Rescue After Major Surgery. *JAMA Netw Open*. Published February 04, 2026. doi:10.1001/jamanetworkopen.2025.55855

Data
Data available: No

Additional Information
Explanation for why data not available: The administrative data used in this study are available from the Swiss Federal Office of Statistics for researchers who meet the criteria for access to the data (contact via gesundheit@bfs.admin.ch). We received these data as agreed in the No 230391 data contract with the Swiss Federal Office of Statistics.
